# Immune aging: biological mechanisms, clinical symptoms, and management in lung transplant recipients

**DOI:** 10.3389/frtra.2024.1356948

**Published:** 2024-02-08

**Authors:** Bhavya Kapse, Marie M. Budev, Jonathan P. Singer, John R. Greenland

**Affiliations:** ^1^ Department of Medicine, University of California, San Francisco, San Francisco, CA, United States; ^2^ Department of Pulmonary Medicine, Respiratory Institute, Cleveland Clinic, Cleveland, OH, United States; ^3^ San Francisco VA Health Care System, Medicine, San Francisco, CA, United States

**Keywords:** immunosenescence, short telomere syndrome, frailty, hypogammaglobulinemia, mitochondrial dysfunction, epigenetic clock

## Abstract

While chronologic age can be precisely defined, clinical manifestations of advanced age occur in different ways and at different rates across individuals. The observed phenotype of advanced age likely reflects a superposition of several biological aging mechanisms which have gained increasing attention as the world contends with an aging population. Even within the immune system, there are multiple age-associated biological mechanisms at play, including telomere dysfunction, epigenetic dysregulation, immune senescence programs, and mitochondrial dysfunction. These biological mechanisms have associated clinical syndromes, such as telomere dysfunction leading to short telomere syndrome (STS), and optimal patient management may require recognition of biologically based aging syndromes. Within the clinical context of lung transplantation, select immune aging mechanisms are particularly pronounced. Indeed, STS is increasingly recognized as an indication for lung transplantation. At the same time, common aging phenotypes may be evoked by the stress of transplantation because lung allografts face a potent immune response, necessitating higher levels of immune suppression and associated toxicities, relative to other solid organs. Age-associated conditions exacerbated by lung transplant include bone marrow suppression, herpes viral infections, liver cirrhosis, hypogammaglobulinemia, frailty, and cancer risk. This review aims to dissect the molecular mechanisms of immune aging and describe their clinical manifestations in the context of lung transplantation. While these mechanisms are more likely to manifest in the context of lung transplantation, this mechanism-based approach to clinical syndromes of immune aging has broad relevance to geriatric medicine.

## 1 Introduction

While human age can be simply formulated as the length of time which a person has been alive, humans change over time in programmed ways. Beyond the well-known progression from childhood to adolescence and from young to elderly adult, we recognize that some people age more slowly than others, hence leading to differences in biological vs. chronological age. Biological age is a more accurate estimate of the functional decline of cells and organs estimated by age-dependent biomarkers including DNA methylation, telomere length, transcriptome and proteomic signatures (1, 2). Certain DNA methylation patterns are so closely linked to biological age that they can be used to define molecular clocks, measuring the organ's effective age. Telomere length is more loosely associated with chronological age, and clinical manifestations of short telomeres are relatively rare.

**Figure 1:**
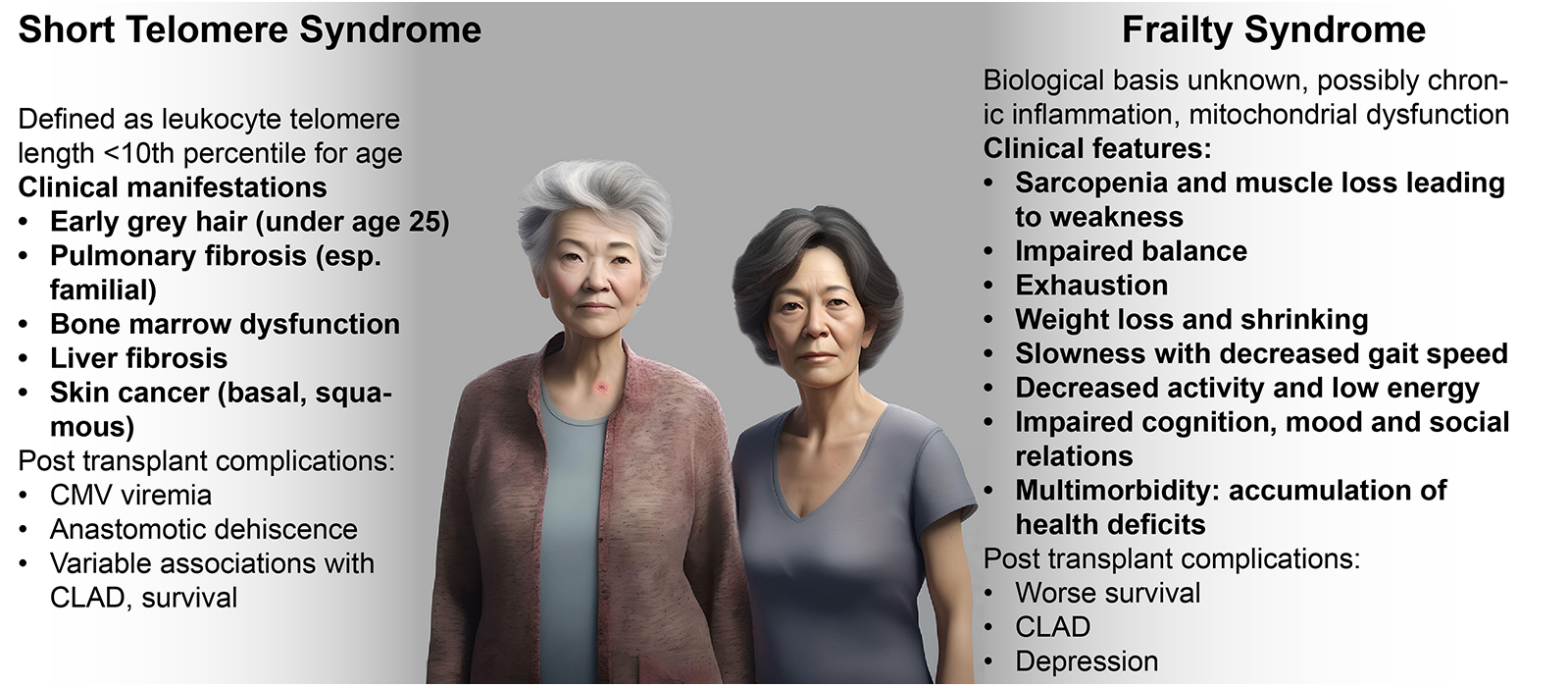
Clinical manifestations of short telomere and frailty syndromes. Clinical characteristics and history may help specific identify age-associated syndromes that may vary in response to therapies. (Image generated using fotor.com AI text to image).

Not everyone ages in the same way. Whereas some individuals develop gray hair from a young age, others may develop wrinkled skin first. Such clinical findings generally reflect biological mechanisms, such as melanocyte senescence with grey hair or a loss of subcutaneous elastin leading to wrinkles, and understanding these mechanisms is key to optimal management of aging syndromes (Figure 1). For example, a clinician recognizing premature gray hair and a compatible family history, might check peripheral blood telomere length and identify a short telomere syndrome, allowing for earlier detection and treatment of pulmonary fibrosis, and helping to preserve quality and duration of life. Age-dependent biological changes may be differentially exacerbated by environmental factors, sleep loss, oncogenic stress, chronic viral infections, diet, and gut dysbiosis. Individual organs may even age at varying rates depending on their respective, genetically-determined biological clocks as well as differential effects of lifestyle and environmental factors (1, 3).

Senescent cells accumulate with age and are fundamental drivers of age-associated pathologies (4). Cellular senescence is characterized by loss of replicative capacity, resistance to apoptosis, and the development of a senescence associated secretory phenotype (SASP), defined by secretome of pro-inflammatory cytokines and chemokines, stem cell stressors, tissue damaging enzymes, and other toxic mediators. Indeed, the use of agents specifically clearing senescent cells (senolytics) or those which modulate their pro-inflammatory secretome (senostatics) have gained attention for their potential in ameliorating age-associated disorders (5). Accelerated senescence may be precipitated by stress signals including DNA damage, telomere erosion, exposure to radiation or oncogenic stimuli, nutrient starvation, and oxidative and genotoxic stress.

The aging immune system is characterized by cellular senescence, with highly proliferative naïve lymphocytes being replaced by more quiescent effector and memory cells in older individuals. Aged T cells display reduced T cell receptor diversity, a more rigid T helper phenotype, and SASP (6). Age-dependent remodeling also significantly affects the phenotype of B cells as well as different subsets of innate cells (7, 8). The primary drivers of immunosenescence include telomere dysfunction, thymic involution, mitochondrial dysfunction and perturbed proteostasis. Cumulative exposure to infectious agents, environmental pollutants, or carcinogens leads to high replicative demands, which can accelerate senescence (9, 10). Immunosenescence compromises protective responses necessary to control microbes, viruses, and tumors, while impairing regulatory mechanisms that prevent autoimmune diseases. Thus, immune aging drives immunodeficiency and a chronic sterile low-grade inflammation referred to as inflammaging.

Distinct molecular and biological drivers of immunosenescence can be associated with specific clinical manifestations, such that recognizing molecular aging phenotypes may help personalize health care within the aging population. These phenotypes linked to defined biological mechanisms can be termed endotypes.

Immune-related endotypes are particularly relevant in lung transplant candidates and recipients. The median age of lung transplant recipients continues to increase, rising from 46 to 60 years over the past 20 years (11). Further, telomere dysfunction is a common indication for lung transplant, a surgery that addresses pulmonary but not immune sequelae of this systemic process. Finally, lung transplant is a profound physiological stress that can unmask aging biology. Table 1 summarizes biological manifestations of immune aging and corresponding treatment proposals in the lung transplant population. While this review focuses on immune aging biological mechanisms and their clinical manifestations in lung transplant recipients, these observations are broadly relevant to gerontology.

**Table 1:** Examples of possible immune aging biology-based treatment strategies.

Primary mechanism	Biological phenotype	Clinical manifestations	Treatment approaches	References
Telomere dysfunction	DNA damage response SASP Replicative senescence	Idiopathic pulmonary fibrosis Bone marrow dysfunction. CMV reactivation	Tailored immunosuppression and infection prophylaxis. Transfusion and bone-marrow stimulants	(12–18)
Preceding CMV infection	Inflation of memory T cells Impaired donor-specific responses	Infection susceptibility CMV reactivation Graft dysfunction	CMV prophylaxis and aggressive treatment	(19–21)
Thymus involution	Reduced TCR repertoire	Increased risk of cancer and infection	Vaccination and infection prophylaxis	(22–26)
Impaired bone marrow hematopoiesis	B-cell dysfunction	Hypogammaglobulinemia Impaired vaccine responses	IVIG Alternate vaccination strategies	(27–30)
Mitochondrial dysfunction	Increased ROS generation Immune activation (e.g., IL-6)	Sarcopenia Frailty	Structured exercise programs Myostatin inhibitors Protein supplementation IL-6 antagonists	(31–37)

## 2 Biologic mechanisms of immune aging

### 2.1 Telomere dysfunction

Telomeres are ribonucleoprotein structures at the end of chromosomes comprised of tandem repeats of TTAGGG sequences and a shelterin protein complex. During cell division, the DNA at the chromosome terminus is not entirely copied due to defects in DNA replication machinery, resulting in single-stranded DNA overhangs. The shelterin complex protects the terminal and single stranded DNA resulting from incomplete replication from being recognized by DNA repair machinery. Telomeres shorten with each round of replication. Telomerase is an enzyme recruited by the shelterin complex which drives *de novo* synthesis of TTAGGG repeats at the telomere ends, thereby maintaining telomere length (38, 39). Telomeres progressively shorten with age and defects in telomere maintenance machinery drive premature ageing phenotypes (40, 41). Short telomeres are an important risk factor for early mortality and result in predisposition to developing age-related disorders including cardiovascular diseases, cancer, and osteoporosis (42–47), whereas, long telomeres may increase the risk of certain cancers, such as melanoma and chronic lymphocytic leukemia (48). Indeed, the heterogeneity in human telomere function may reflect this balance between promoting cellular regeneration and limiting cancer risk.

While telomeres tend to become shorter with cellular replication, physiologic consequences manifest only when telomers reach a critical length. Critically short telomeres no longer bind to Shelterin complexes, leading to uncapping of single-stranded DNA and subsequent recognition by DNA damage response pathways, such as ataxia-telangiectasia mutated (ATM)-dependent activation of p53 and checkpoint kinases (49). These DNA damage responses halt cell cycle progression leading to replicative arrest and development of SASP (50). Clinically, telomere length is generally reported as an average number of base pairs within a cell type. Notably, across cell populations and even within the same cell, telomere lengths have a broad distribution. Thus, the finding that lower mean telomere lengths drive pathology likely reflect that an increased number of cells are approaching the critical limit that will drive senescence.

Telomere shortening may be resulted by genetic factors or senescence-precipitating environmental cues. Mutations in telomere maintenance machinery or defects in DNA repair signaling result in a spectrum of inheritable diseases known as telomeropathies (51). Telomeropathies present with pulmonary fibrosis, liver cirrhosis, aplastic anemia, and sporadic acute myelogenous leukemia; and multiple pathologies may concurrently manifest in the same individual due to the common underlying etiology of telomere dysfunction (52). Telomere shortening is also accelerated by lifestyle factors including obesity, diet and exercise, and smoking. Exposure to harmful substances including pollutants, genotoxic or carcinogenic agents, and radiation also drive telomere shortening (2, 53). The clinical complications of telomere dysfunction are detailed in Section 3.1.

### 2.2 The epigenetic clock

While telomere dysfunction is a well-studied aging mechanism, particularly in the transplant population, it is only one of many biological mechanisms driving aging phenotypes. An important regulator of biological aging is epigenetic age. While there are also age-associated changes in histone modifications and chromatin accessibility, epigenetic clocks are typically defined based on patterns of DNA methylation (54). Cytosine guanine dinucleotides (CpG) in DNA sequences undergo cytosine methylation by DNA methyltransferases (DNMT), generally rendering the DNA regions less transcriptionally active. Conversely, ten-eleven transposition (TET) participate in DNA demethylation. This process of reversibly redacting DNA regions is a large part how cells retain their identities. For example, T cells have a set of specific methylation regions that have been shown to determine regulatory vs. effector functions (55). Secondary to cell type, DNA methylation patterns play an important role in aging phenotypes.

Consistent patterns of DNA methylation changes have been observed across many cell types conserved across species. These sites, linked to long-term histone-based epigenetic silencing, are associated with development, cancer, obesity, and longevity-related genes (56). Linear combinations of these DNA methylation patterns can allow construction of epigenetic clocks that are strongly associated with cell age. These patterns likely reflect imperfections in the epigenetic maintenance system, and these clocks have meaningful age acceleration in response to environmental stressors. Epigenetic clocks include Horvath's clock, Hannum's clock, DNA PhenoAge, and DNA GrimAge (54, 57). These clocks differ in their training and tuning to target either time since birth, rate of aging, or time to death. Schitzophrenia, for example, primarily accelerates mortality clocks, like GrimAge (58). Mood stabilizers and antipsychotics are associated with decreasing epigenetic age, and dietary interventions to improve longevity have been shown to decrease GrimAge (59). It remains to be seen whether therapies that selectively modify epigenetic age would have a corresponding effect on mortality (60).

Divergence between chronologic and biologic age, as measured by epigenetic clock, can be relevant in transplant recipients. In a cohort of renal transplant recipients, peripheral blood DNA methylation age was a better predictor than chronologic age of infections in the first year (61).

### 2.3 Senescence in the adaptive immune system

#### 2.3.1 Thymic involution

The thymus is a bilobed organ in the anterior mediastinum that is the primary site for maturation of T cells. The cellular composition of thymus includes thymocytes (immature T cells) and thymic epithelial cells, which play a role in negative selection of alloreactive cells. T cells released by thymus are immunologically naïve until they recognize an antigen with high affinity. Antigen recognition together with costimulatory signals leads to their proliferation and differentiation into effector cells. A subset of effector cells survives long-term to provide immunological memory to the specific antigen. Thymus function declines with age, otherwise known as age-related thymus involution. Thymus involution leads to reduced proportions of thymocytes and thymic epithelial cells, increased perivascular space, and adipose tissue deposition in thymic compartments. Disruption of thymic architecture results in loss of naïve T cells and a relative increase in memory T cells, thereby leading to reduced T cell-capacity of recognizing diverse pathogen-derived antigens leading to increased susceptibility to infection (6). Thymic involution may also explain decrease in immune responses to vaccines (62).

T cell maturation requires T cell receptor rearrangement and results in the formation of T cell receptor excision circles (TRECs) (63, 64). TRECs are stable and non-replicative, and progressively diminish with cell divisions. TRECs are therefore a useful measure of thymic output. Recent thymic emigrants are identified by elevated copy numbers of TRECs, and TREC frequency declines with aging (22, 23). Studies have correlated loss of transplant recipient thymic function, as measured by decreased TREC frequency, with poor post transplantation outcomes including cancer (24) and viral infections (25). Pre-transplant thymic function is identified as independent risk factor for viral infection post solid organ transplantation (26).

Strategies to address thymus loss could potentially have a role in transplantation. Grafting thymus lobes from an allogeneic donor into the neonatally thymectomized mice induced tolerance towards tissue antigens of the donor (65–67). Hence, concurrent thymus transplantation is a possible strategy to induce allospecific tolerance, while preserving immune function. Strategies to reverse thymus involution might also use thymostimulatory cytokines (68).

#### 2.3.2 T cell dysfunction

Cumulative antigenic exposure results in accumulation of terminally differentiated pro-inflammatory T cells with aging. Aged T cells display senescence hallmarks including short telomeres, DNA damage responses, replicative arrest, resistance to apoptosis, and secretion of pro-inflammatory cytokines (6). T cell aging is accelerated by chronic viral infections such as CMV, which drives the expansion of terminally differentiated CD8+ effector memory cells (T_EMRA_) (69). T_EMRA_ cells exhibit markers of senescence while retaining the cytokine production and cytotoxicity potential of precursor cells (19). T_EMRA_ cells may have greater potential to drive allograft damage, as increased proportions of circulating T_EMRA_ correlate with higher risk of renal graft failure (20). In lung transplant recipients, CMV infection increases the risk of graft failure and susceptibility to infections (21). Hence, age-associated T cell defects driven by persistent viral antigenic stimulation may worsen clinical outcomes in transplantation recipients.

#### 2.3.3 Regulatory T cell dysfunction

Tregs are professional anti-inflammatory cells that have a critical role in limiting allograft rejection (70). Aging correlates with a rise in inflammatory T cells at the expense of Tregs, The preponderance of data link robust Treg responses to improved outcomes, with the caveat that acute rejection drives a compensatory Treg expansion, limiting their utility as a biomarker (71). Tregs in older recipients transition to an effector phenotype in the context of IL-6 stimulation, a cytokine associated with senescent cells, Age-associated inflammation further drives dysregulation of microbiota in aged lungs perpetuating an inflammatory phenotype (72). Accordingly, therapeutic targeting IL-6, restoration of lung eubiosis by heathy diet and exercise harnessing the gut-lung axis, or directly administering *in vivo* expanded Treg have been proposed as strategies to improve graft survival. Notably, the limited proliferative capacity of Tregs from transplant recipients has proven a critical barrier to their development as a therapeutic (73).

#### 2.3.4 B cell dysfunction

B-cells and plasma cells generate antibodies in concert with T cell help. Such antibodies are key to immune responses to infections, but also drive antibody-mediated rejection. B cell antigen specificity and immunoglobulin type are refined through DNA rearrangements, known as class-switch recombination, which involves exchanging the IgM or IgD heavy chain region of the antibodies to an IgA, IgG, or IgE type, resulting in improved ability to eliminate pathogens, and somatic hypermutation, involving DNA mutations that generate antibodies with high antigen affinity. However, B cell precursors derive from the bone marrow, and an age-dependent shift in bone marrow myelopoiesis leads to impaired generation of B cell precursors (74). Extrinsic and cell intrinsic factors necessary for commitment, differentiation and maintenance of mature B cells are reduced with age (75, 76). Antibodies produced by aged B cells are less potent in class switching and somatic hypermutation (77), which may manifest as poor vaccination outcomes in elderly (7). As part of age-associated inflammation, B cells displaying a hyperreactive pro-inflammatory phenotype accumulate with age (78). Aged B cells respond in a B cell receptor independent innate fashion and contribute to inflammation and disease pathogenicity in autoimmune and autoinflammatory disorders (78).

The inflammatory phenotype of aged B cells may reduce allograft tolerance, as depletion of B cells delayed allograft rejection in aged mice and adoptive transfer of aged B cells worsened graft survival in young mice in a model of skin transplantation (79). However, an understanding the role of aged B cells in regulating outcomes in lung transplantation remains nascent.

### 2.4 Aging in the myeloid lineage

#### 2.4.1 Macrophages and dendritic cells

Lung-resident alveolar macrophages exhibit a dual role in modulating inflammation. At steady state, alveolar macrophages clear apoptotic debris and subdue inflammation. These anti-inflammatory macrophages play a protective role in solid organ transplantation. For example, they promote the resolution of inflammation resulting from ischemia-reperfusion injury (80, 81). However, upon sensing of pathogens or tissue debris, alveolar macrophages are activated to release proinflammatory mediators which promote tissue injury (8, 82). Aging leads to impaired apoptotic clearance and anti-inflammatory cytokine production by macrophages thereby leading to prolonged inflammation and tissue injury (8, 83). Reduced reparative functions of aged alveolar macrophages potentiates inflammation potentially impairing graft survival.

Similarly, dendritic cells display an age-associated decline in function, evidenced by reduced phagocytosis, impaired antigen processing and presentation, reduced migration potential, diminished expression of co-stimulatory molecules and impaired cytokine production, leading to a dampened adaptive response in aged individuals and altered alloimmune responses in solid organ transplantation (6, 8).

#### 2.4.2 Myeloid derived suppressor cells and dendritic cells

Myeloid derived suppressor cells (MDSCs) are a heterogenous cell population of myeloid lineage with potent immunosuppressive activity. MDSCs are pathologically activated in inflammation, infection, and cancer. Aging skews the differentiation of hematopoietic progenitor cells towards myeloid lineage resulting in reduced lymphopoiesis and correspondingly increased proportions of myeloid derived suppressor cells (MDSCs). MDSCs inhibit T cell proliferation and are associated with improved graft survival in models of solid organ transplantation (84). Aged MDSCs express increased levels of cell cycle-associated senescent markers and can suppress T cells (85), potentiating their beneficial role in suppressing alloimmunity in solid organ transplantation.

### 2.5 Mitochondrial dysfunction

Mitochondria are cellular organelles responsible for the synthesis of cellular ATP and are thus considered the powerhouse of the cell. Mitochondrial dysfunction is a well-known hallmark of age-associated senescence. Deletion of genes encoding proteins involved in mitochondrial function also promotes T cell dysfunction and leads to the acquisition of a pro-inflammatory phenotype (86, 87).

Mitochondrial ATP synthesis involves the Krebs cycle and oxidative phosphorylation. The Krebs cycle utilizes the catabolic end products of sugars, fatty acids, and proteins to generate substrates for oxidative phosphorylation (88). Electrons from TCA cycle derivatives are transferred sequentially to the respiratory chain complexes and eventually to molecular oxygen. The electron transfer is coupled with the release of protons into inter membrane space leading to the formation of an electrochemical gradient which is subsequently resolved by the ATP synthase, which couples the restoration of protons back to the matrix with ATP synthesis (89). Senescent cells are less efficient in ATP generation due to reduced mitochondrial membrane potential and impaired oxidative phosphorylation (90).

Reactive oxygen species (ROS) are a major by-product of ATP synthesis. Premature leak of electrons from respiratory chain complexes results in partial reduction of O_2_. The O_2_^−^ free radical is rapidly dismutated to H_2_O_2_ by enzymatic actions and enters the cytosol to convey signaling pathways involved in cell proliferation, differentiation, and apoptosis (91–93). Senescence is associated with increased ROS production (31). Conversely, mitochondrial ROS are important drivers of cellular senescence. ROS production caused by dysfunctional mitochondria can drive oxidative DNA damage leading to cell cycle arrest (94). Furthermore, ROS lead to the creation of γH2AX foci, histone triggers of the DNA damage response that drive a senescence phenotype (95). ROS are also potent inducers of telomere dysfunction and can result in replicative senescence. Experimentally, ROS promote hepatocyte damage in a model of liver ischemia reperfusion injury (96). ROS scavenging leads to rescued telomere shortening in CD8+ T cells and extended lifespan in fibroblasts cultured *in vitro*.

#### 2.5.1 Mitochondria in frailty, sarcopenia, and immunosenescence

Inherited disorders of mitochondrial function primarily manifest with myopathies, or disorders of skeletal muscle. Mitochondrial dysfunction can also manifest as encephalomyopathy, neuromuscular disorders, cardiac arrhythmias, diabetes, loss of vision and hearing, and stunted growth. The age-related loss of skeletal muscle, termed sarcopenia, is clinically defined as pathologically reduced muscle mass and strength and considered distinct from atrophy secondary to inactivity. Sarcopenia increases the risk of falls, fractures, physical disability, and death (97, 98). Sarcopenic muscle is characterized by increased mitochondrial DNA mutations, leading to the synthesis of dysfunctional respiratory chain components with reduced ATP synthesis capacity and increased ROS production (32). Physical exercise is potentially the most effective treatment strategy for sarcopenic individuals (99). Although the effect of exercise on sarcopenia is more complex than just muscle activation, exercise is associated with improved muscle mass, strength, and physical performance in older adults (33). Restoring metabolism of aged myeloid cells by targeting lipid signaling pathway may help decrease age-associated inflammation and restore mitochondrial homeostasis (100). Alternatively, controlled physical exercise promotes generation of ROS (101, 102) and simultaneous upregulation of antioxidant enzymes which counteract ROS generation (103). Exercise may help reduce the effects of oxidative stress in advanced age and thereby delay the onset of sarcopenia in physically active elderly population.

Older adults with sarcopenia have increased circulating cell-free mitochondria DNA (104). Stressed cells release endogenous contents that are recognized by pattern recognition receptors (PRRs) present on antigen presenting cells, induce an inflammatory response. Such endogenous moieties are referred to as danger associated molecular patterns (DAMPs). Mitochondrial DNA is capable of functioning as a DAMP when released into the cytosol as well as the circulation. Cell free mitochondrial DNA induces pro-inflammatory pathways to promote IFN-β release (105, 106). Furthermore, high levels of mitochondrial DAMPs in circulation are associated with increased severity of primary graft dysfunction, i.e., severe acute lung injury in first 72 h of transplantation which is a major cause of mortality in lung transplant recipients (107). Inhibition of receptors sensing damaged mitochondrial components rescued graft injury in a mouse orthotopic lung transplant model of primary graft dysfunction (34).

Systemic accumulation of ROS in sarcopenic patients may potentially contribute to immune activation (108). In a model of skin transplantation, mice undergoing regular exercise had improved graft survival (109). Interestingly, prolonged and exhaustive exercise promotes endotoxemia and systemic inflammation (110, 111). Thus, structured exercise interventions are worth investigating to address immune activation from mitochondrial dysfunction.

Beyond the role of systemic mitochondrial dysfunction in immune stimulation, dysfunctional mitochondria can directly drive immunosenescence through lymphocyte and myeloid cell dysregulation. Specifically, mitochondrial activities, including glycolysis and oxidative phosphorylation are implicated in T and B cell activation, differentiation, and proliferation, and mitochondrial impairment leads to a shift towards myeloid proliferation (112).

## 3 Clinical manifestations and approaches to immune aging

### 3.1 Clinical complications of short telomere syndrome post lung transplantation

As described above, telomere length is genetically determined with a defined normal range, but germline or somatic mutations in telomerase maintenance genes can increase the rate of telomere shortening that usually occurs with aging, leading to premature aging syndromes. The short telomere syndrome (STS) refers to a constellation of clinical manifestations caused by mutations in the telomerase and other telomere maintenance genes. The STS presents as degenerative clinical phenotypes of organ failure across multiple organ systems. The adult clinical presentation of the STS phenotype can include pulmonary fibrosis, with usual-interstitial pneumonia pattern pulmonary fibrosis being the most common pulmonary manifestation of STS; emphysema and other lung diseases; cryptogenic cirrhosis and hepatopulmonary syndrome; immunodeficiencies; bone marrow suppression or failure; bone marrow derived malignancies such as myelodysplastic syndrome (MDS) and acute myeloid leukemia (AML); and other malignancies including squamous cell cancers (113). Clinical features and presentations that can heighten the suspicion for STS are contrasted with frailty syndrome in Figure 1 (114).

Telomere length measurement has become routine practice in some clinical Interstitial Lung Disease (ILD) programs but has not been integrated in the assessment of lung transplant candidates with IPF at most lung transplant centers in the US. Further, uniformity in standardization of testing is still not established (113). The threshold of age-adjusted lymphocyte telomere length the <10th percentile has been examined as a predictor of worse outcomes in IPF patients receiving immunosuppression. Age adjusted telomere lengths <10th percentile of normal (shortest telomere lengths) can be found in 40% of patients with familial IPF and 25% of sporadic cases without a family history of fibrosis.

The implications of telomere length in IPF lung transplant recipients have been of growing interest. STS has been reported to be twice as common in IPF lung transplant recipients comparted to those with IPF who do not receive transplantation (115). In one cohort, 32% of IPF lung transplant recipients had a telomere length <10th percentile (116). Questionnaire-based screening followed by comprehensive telomere testing identified critically short (<1st percentile or known pathological variant) in 8% of ILD transplant candidates (117). Its frequency may justify establishing the diagnosis of underlying STS prior to lung transplantation in effort to anticipate and prevent the post-transplant morbidities and complications described below. Identification of short telomeres may allow strategies to prevent irreversible complications of STS in lung transplant recipients (113, 115, 116). Table 2 provides a suggested multidisciplinary team evaluation of STS in the pre or post lung transplantation (114).

**Table 2:** Multidisciplinary team framework of care Pre or post lung transplant recipients With short telomere syndrome (113, 114).

• Consult hematology to address treatment and evaluation of cytopenias and need for bone marrow biopsy • Consult genetic counseling to assist with familial risk for short telomere syndrome risk. • Consult hepatology for liver parenchyma evaluation and appropriate imaging for evidence of cirrhosis, evaluation for hepatopulmonary syndrome and need for liver transplantation. • Consult Infectious Disease for management of antimicrobial and antiviral prophylaxis in setting of cytopenias • Routine surveillance skin checks for assessment of skin cancers • If lung transplant candidate with STS is CMV IgG negative and stable, favor a CMV negative donor if feasible.

#### 3.1.1 Graft-specific complications

Comparisons of STS lung transplant recipients and those with normal telomere lengths have shown inconsistent results in terms of survival and graft outcomes such as primary graft dysfunction (PGD), acute cellular rejection (ACR), and CLAD. Newton et al. reported that STS lung transplant recipients had an increased rate of PGD and CLAD as well as with a nearly 11-fold increased risk of death compared to non-STS referents (116). Similarly, Courtwright et al. found that pre-transplant shortened telomere length was associated with leukopenia and decreased CLAD-free survival after lung transplant (118). Faust et al. found decreased telomere length was associated with leukopenia after transplant but was not associated with worse CLAD-free survival (12). In contrast, a more recent study of IPF lung transplant recipients found that telomere mutations and length was not associated with PGD, ACR, time to CLAD, or post-transplant survival (115). One potential reason for these inconsistencies is that short telomeres were not defined in the same way across all studies.

T-cell immunosenescence may be the predominant reason for the observation of less frequent acute rejection in STS lung transplant recipients. In a cohort of STS lung transplant recipients, there was an age-dependent decrease in acute rejection associated with evidence of immune aging, which was manifest as T cell differentiation, clonal expansion, and exhaustion (119). A similar immunosenescent phenotype has been observed in older renal transplant recipients, with short telomeres, evidence of T cell exhaustion, and decreased rates of acute rejection (120). IPF lung transplant recipients have relatively suppressed donor-specific effector and regulatory T cell responses at the time of transplant. This type of immunesenescence results in a state of anergy that is readily broken with strong cytokine stimulation, such as would be seen with infections (121). Thus, while lower rates of acute graft rejection may be seen in STS transplant recipients, these recipients are at continued risk of chronic rejection (122, 123).

#### 3.1.2 Bone marrow suppression

Bone marrow reserves in STS lung transplant recipients are vulnerable. Cytopenias and bone marrow complications can emerge in the setting of antimetabolite or other immunosuppression medications, and certain antimicrobial and antiviral prophylaxis (124). Short recipient telomere length is a risk factor for clinically significant leukopenia (125). In a cohort with IPF, those who received lung transplantation were more likely to have bone marrow dysfunction requiring bone marrow biopsy (4% vs. 29%) (126). Management strategies to avert or mitigate bone marrow suppression may include dose modification of standard immunosuppression regimen by holding or decreasing antimetabolite therapies, holding viral polymerase inhibitors like valganciclovir, and using dapsone in place of trimethoprim/sulfamethoxazole for *Pneumocystis* prophylaxis (114).

Other than reduction of immunosuppression therapies and avoidance of myelosuppressive antimicrobial and antiviral prophylaxis, treatment for post-transplant cytopenia or bone marrow suppression is mainly supportive. Blood or platelet transfusions and granulocyte colony stimulating factor (GCSF) for neutropenia have been utilized. GCSF use has not been shown to increase risk for acute cellular rejection or CLAD when used in severely neutropenic patients (127). There is hesitation to use danazol in this population is due a lack of data and potential risk for MDS and AML (113). Bone marrow biopsy may be necessary to evaluate dyscrasias in certain cases given the risk for MDS and AML (126). Therefore, a multidisciplinary team including hematologists should be considered in the care of STS lung transplant recipients.

#### 3.1.3 CMV and other herpes viral infections

STS lung transplant recipients are at higher risk for herpes virus (HSV) and cytomegalovirus (CMV) infections due to T-cell immunodeficiency. Indeed, STS lung transplant recipients have up to a 5-fold increased risk for relapsing CMV viremia (13, 114, 128). As CMV viremia is a risk factor for CLAD, this telomere-dependent impairment of T cell immunity may contribute to increased CLAD risk in STS lung transplant recipients (43, 129). At the same time, several factors can complicate the treatment of CMV viremia in the STS population. STS lung transplant recipients are susceptible to CMV drug resistance and can develop myelosuppression from antiviral medications including ganciclovir and valganciclovir. Alternative treatments with foscarnet or cidofovir have limited utility in certain situations due to renal and bone marrow suppression and poor tolerance. Letermovir, a viral terminase inhibitor, has been approved for the prophylaxis of CMV infection in allogenic stem cell transplants. Letermovir has been studied off label for CMV prophylaxis in thoracic transplant recipients, including 37 lung transplant recipients, in whom it was well tolerated and effective. Without the side effect of bone marrow suppression, letermovir use may have facilitated antimetabolite therapy in certain number of patients (130). While there is concern that letermovir may be susceptible to CMV mutations conferring resistance, further study is warranted to determine whether letermovir could have a role for CMV prophylaxis, specifically in STS lung transplant recipients.

Iasella *et al*. (131) reported an increased risk for Epstein-Barr virus (EBV)-associated posttransplant lymphoproliferative disorder (PTLD) in IPF lung transplant recipients, which was postulated to be secondary to STS immune dysfunction (131). When designing care protocol approaches to patients with known STS, consultation with infectious disease providers may be helpful to consider alternative agents to prevent and manage herpes viral infections (113).

#### 3.1.4 Liver disease and cirrhosis

Liver abnormalities have been reported in approximately 10% of STS patients. The combination of IPF, liver disease and bone marrow failure is a classic triad associated with severe STS (132). Gorgy *et al*. demonstrated that telomere shortening can also be linked to non-cirrhotic portal hypertension manifested as hepatopulmonary syndrome (HPS). STS patients with progressive HPS can present with clinical manifestations of severe hypoxemia, development of portal hypertension and complications including gastrointestinal bleeding from varices. Liver transplantation can reverse the hypoxemia in HPS however STS patients remain at risk for the development of progressive IPF (133). Several case reports describe combination of liver and lung transplantation in STS patients with end-stage IPF and cirrhosis with acceptable early outcomes (134, 135). Early screening for liver pathologies is recommended in patients with diagnosed STS in the pre-lung transplant period with appropriate laboratory evaluation, imaging, and subsequent referral to a hepatologist if liver disease or cirrhosis is suspected. Continued surveillance and monitoring for liver disease should be continued post transplantation.

#### 3.1.5 Cancer risk

Lung transplant recipients are at significantly increased risk of malignancy. Only 76% of double lung transplant recipients and 66% of single lung transplant recipients are free from non-skin malignancies by 15 years post-transplant, with a notable burden of lung cancer in single lung recipients (11). STS does not necessarily increase the risk of developing cancer in this cohort (116), but chronic immunosuppression appears to amplify the cancer risk associated with immune aging. Epigenetic dysregulation in combination with accumulation of DNA damage and senescent cells contribute to oncogenesis in the aged population, while proinflammatory cytokines can support tumor growth and metastasis (136).

Although the overall risk for cancer in STS is relatively low, IPF and STS can be associated with certain malignancies. IPF lung transplant recipients have increased risk for EBV-associated post-transplant lymphoproliferative disease, particularly after receiving alemtuzumab, in induction agent associated with immunosenescence (131). Outside of transplant, individuals with STS have increased risk for myelodysplastic syndrome (MDS) and acute myeloid leukemia (AML). STS patients who develop MDS or AML may have concomitant lung fibrosis or liver disease or both making treatment decisions complex. The option for hematopoietic stem cell transplant and immunosuppressive regimens may be difficult due to risk for pulmonary injury and the choice for living related donor given the familial nature of STS may be limited (137). Combined bone marrow and lung transplantation has been reported with STS-associated bone marrow failure and ILD (138).

In sum, telomere length may be a potential biomarker pre-transplantation to assess the risk for various post-transplant complications, but the limited and inconsistent evidence from a series of single center studies must approached with caution. There is insufficient evidence to exclude lung transplant candidates from listing based on the presence of STS categorically, but there may be some circumstances where the severity of liver and/or bone marrow involvement may preclude the potential benefits of lung transplantation.

### 3.2 Donor immune age and its impact on the allograft

The airway epithelium plays an essential role in detecting pathogens and initiating immune responses. However, the lung epithelium is donor derived so its age is determined by the donor date of birth. This can be demonstrated by epigenetic measurements of age, where airway epithelial cell age one-year post-transplant is strongly correlated with donor age but has no association with recipient age (139). Decreasing telomere length in donor peripheral blood cells has been associated with worse CLAD-free survival in one cohort (12). Despite a plausible mouse model demonstrating how short club cell telomeres lead to CLAD pathology (140), other studies have not replicated this finding (121, 141). Telomere dysfunction is sufficiently rare in the lung donor population that we would not recommend screening donors.

Even if the immune age of the allograft is normal at the time of transplant, post-transplant complications may accelerate biological mechanisms of lung aging, with potential clinical implications. There is a substantial turnover in epithelial cells following lung transplant, as evidenced by high levels of donor derived cell-free DNA (142) and diffuse Ki-67 positivity in some airways (14). PGD is associated with both increased airway epithelial epigenetic age (139) and accelerated telomere dysfunction (14). Epigenetic remodeling from PGD is linked to persistent activation of hypoxia signaling, while telomere dysfunction in airway epithelial cells is associated with inflammation and dysregulated remodeling. Thus acceleration of epithelial aging pathways may be a mechanism linking PGD to CLAD risk (14).

### 3.3 Hypogammaglobulinemia

Hypogammaglobulinemia is a symptom of B and plasma cell dysfunction that manifests more commonly at extremes of age. Primary hypogammaglobulinemia can reflect inherited immunodeficiency, whereas secondary hypogammaglobulinemia may be a side effect of immunosuppressing medications. In practice, it may not be clear whether hypogammaglobulinemia is primary or secondary or a combination. Studies have reported a gradual decrease in immunoglobulin G (IgG) and IgM concentrations with aging, with IgG levels decreasing considerably between the third and sixth decade of life (27).

Hypogammaglobulinemia is a well described phenomenon in adult lung transplant recipients. In these recipients, IgG levels are inversely correlated with bacterial infections, invasive fungal infections, days in the hospital, and CLAD risk (28, 29). Severe hypogammaglobulinemia defined as IgG level <400 mg/dl was associated with CLAD, increased antibiotic use, and bacterial, fungal and CMV reactivation (30). IgA, IgM, and IgG levels may decrease post lung transplantation, and immunoglobulin level screening may be considered before and after transplant to identify patients who may need IgG replacement therapy post lung transplantation to reduce infection risk. Hypogammaglobulinemia can be accompanied by suppression of vaccine responses. Completing the recommended vaccinations prior to transplantation is important aspect of bacterial and viral prophylaxis since multiple immunosuppressive agents administrated post-transplant can further attenuate vaccine responses (29). Consultation with a clinical immunologist may be helpful to design protocols and manage recipients with hypogammaglobulinemia to consider immunoglobulin replacement and vaccine protocols.

### 3.4 Impaired vaccine responses

Vaccine responses in transplant recipients may be diminished by immunosenescence, inflammaging, and comorbid disease states (62), all of which are compounded by post-transplant immune suppression. Less than 20% of transplant recipients generated expected responses to the SARS-CoV-2 mRNA vaccine in one study (143). Relative to other solid organ transplant recipients, lung transplant recipients have the most impaired/blunted vaccine responses, for instance, there were 68% patients which did not respond to vaccines in a separate study (144).

Several strategies have been proposed to address poor vaccine responsiveness in transplant recipients. Whenever possible, transplant candidates should receive necessary vaccines prior to transplantation. For recipients transplanted prior to the COVID-19 pandemic, this was not possible, and so a common strategy was to provide additional boosters until acceptable antibody titers were achieved. However, some recipients did not display evidence of immunity despite several boosters. Infusion of virus specific monoclonal antibodies has been used (145), although this strategy has not been consistently effective and can be expensive and susceptible to viral evolutionary escape. As antimetabolites are most strongly associated with poor vaccine responses, a three week pause in antimetabolites around the time of vaccination has been proposed. However, it is not known to what extent this pause could trigger initial or progressive chronic rejection (146). Other theoretical approaches to improving vaccine doses include using higher antigen doses or novel adjuvants, although there are diminishing returns to additional antigen loads (147). Immune responses vary by vector and route of administration (148), and these approaches have not been well studied in transplant recipients. Other interesting ideas include using senolytics or immune modulatory drugs like mTOR inhibitors, or adjusting vaccine patterns based on circadian rhythms (62).

### 3.5 Immune dysregulation leading to frailty

Frailty is a clinical syndrome characterized by weakness, muscle loss, fatigue, and impaired physical activity. Frailty refers to a general depletion in physiologic reserves resulting in disproportionate decline in health status in response to an otherwise inconsequential stressor (149). Frailty is linked to delisting and death in lung transplant candidates, and predicts worse quality of life and survival post transplantation (62, 150). Intriguingly, incident frailty after lung transplantation is also associated with increased risk for CLAD, suggesting that frailty may be associated with systemic inflammation that can drive chronic rejection (151). A pro-inflammatory subtype of frailty has been identified, with increased levels of IL-6, CRP, PTX3, TNF-R1, and IL-1RA, mitochondrial stress, sarcopenia, malnutrition, and decreased exercise tolerance (152). Age-adjusted short leukocyte telomere length has also been associated with frailty in some cohorts (153). As frailty can be observed even in younger transplant candidates and recipients, there is increasing interest in quantifying frailty to stratify transplant risk and identify individuals for frailty-specific interventions. Frailty is a potentially modifiable risk factor, as exercise programs can improve at least some components of frailty (154). Exercise has been shown to impact immune function, boosting NK and T cell proliferation and cytotoxicity, in particular (155). However, the impact of structured exercise programs on immunosenescence and inflammation in transplant needs further study.

### 3.6 Immunosuppression intolerances

Immunosuppressive medications are relatively more potent in older recipients. Due to alternations in the metabolism of drugs through CYP3A5-enzyeme complexes, similar doses of calcineurin inhibitors (CNI) achieve greater blood levels in older patients (156, 157). The clearance of steroids may also decline with age (156–158). Further, mouse and human studies show that tacrolimus is more potent in older immune systems, relating to suppressed calcium influx and intracellular calcineurin levels (159). There is a paucity of clinical trial evidence comparing immunosuppression approaches in older patient cohorts. In clinical practice, responses to immune aging-related side effects, such as leukopenia or infection, tends to be responsive rather than anticipatory.

As an alternative or adjunct to a CNI-based regimen, mTOR inhibitors have been considered to address CNI-based complications, including renal dysfunction and malignancy. The 4EVERLUNG study combined low-dose CNI and mTORi at 3–18 months post-transplant for a 4-drug immune suppression regimen. At 5-year follow up, 46% of participants had discontinued the 4-drug regimen, there was a trend towards improved CLAD-free survival, but there were no significant differences in long-term outcomes. In animal studies, the mTOR inhibitor rapamycin increases longevity in invertebrates and promotes allograft survival in old mice. Further, low daily doses of a mTOR inhibitor for 6 weeks before administration of influenza vaccine to older adults was shown to increase the antibody response to the vaccine by 20% (160). Whether there is a role for mTOR inhibitors in addressing immune aging in lung transplant recipients remains to be seen, but investigating such biological mechanisms may be necessary for clinical success in such studies.

## 4 Conclusions

Multiple mechanisms drive immune aging, and immune aging phenotypes may have clinically relevant manifestations. Early identification of biological manifestations of aging is important. Because organ systems typically have impressive physiological reserve, biological aging processes begin long before they manifest as physiological phenotypes, and even further before they result functional decline.

Lung transplantation is of particular interest in aging biology for several reasons: STS is an indication for lung transplantation, such that this otherwise rare aging syndrome is highly enriched in the lung transplant population. Lung transplant candidates tend to be older than for other solid organs and they have endured physiologic stresses that may drive age acceleration. Further, potent alloimmune responses necessitate higher levels of immunosuppression, and transplantation itself is a major physiologic stressor that can unmask age-associated pathologies. Although we are gaining experience managing aging transplant recipients, some have questioned the ethics of transplanting older individuals based on the decreased utility in terms of expected survival and the principle of justly granting an “equal chance to live a full life” (161).

Accordingly, lung transplant recipients are particularly sensitive to immune aging complications. Immune aging can be driven by telomere dysfunction, CMV infection, thymus involution, impaired bone marrow hematopoiesis, and mitochondrial dysfunction, and the degree to which these mechanisms contribute may contribute to the variety of manifestations of immune aging syndromes. While STS is the best-defined immune aging syndrome in this population, lung transplant recipients are also at risk for hypogammaglobulinemia and or immune aging from mitochondrial dysfunction. There are multiple interventions under investigation to address immune senescence mechanisms, including danazol for STS, senolytics to remove senescent immune cells, and exercise and nutrition to address mitochondrial dysfunction. There is a compelling rationale to target investigations of treatment strategies for immune aging mechanisms based on biological phenotypes. However, much work remains to define and match appropriate interventions to endotypes of immune age.

## References

[1] Nie C Li Y Li R Yan Y Zhang D Li T Distinct biological ages of organs and systems identified from a multi-omics study. Cell Rep. (2022) 38(10):110459. 10.1016/j.celrep.2022.110459 35263580

[2] Shammas MA. Telomeres, lifestyle, cancer, and aging. Curr Opin Clin Nutr Metab Care. (2011) 14(1):28–34. 10.1097/MCO.0b013e32834121b1 21102320 PMC3370421

[3] Xu W Wong G Hwang YY Larbi A. The untwining of immunosenescence and aging. Semin Immunopathol. (2020) 42(5):559–72. 10.1007/s00281-020-00824-x 33165716 PMC7665974

[4] López-Otín C Blasco MA Partridge L Serrano M Kroemer G. The hallmarks of aging. Cell. (2013) 153(6):1194–217. 10.1016/j.cell.2013.05.039 23746838 PMC3836174

[5] Kang C. Senolytics and senostatics: a two-pronged approach to target cellular senescence for delaying aging and age-related diseases. Mol Cells. (2019) 42(12):821–7. 10.14348/molcells.2019.0298 31838837 PMC6939651

[6] Mittelbrunn M Kroemer G. Hallmarks of T cell aging. Nat Immunol. (2021) 22(6):687–98. 10.1038/s41590-021-00927-z 33986548

[7] Frasca D Diaz A Romero M Garcia D Blomberg BB. B cell immunosenescence. Annu Rev Cell Dev Biol. (2020) 36(1):551–74. 10.1146/annurev-cellbio-011620-034148 33021823 PMC8060858

[8] Boe DM Boule LA Kovacs EJ. Innate immune responses in the ageing lung. Clin Exp Immunol. (2016) 187(1):16–25. 10.1111/cei.12881 27711979 PMC5167032

[9] Pandics T Major D Fazekas-Pongor V Szarvas Z Peterfi A Mukli P Exposome and unhealthy aging: environmental drivers from air pollution to occupational exposures. GeroScience. (2023) 45(6):3381–408. 10.1007/s11357-023-00913-3 37688657 PMC10643494

[10] Haynes L. Aging of the immune system: research challenges to enhance the health span of older adults. Front Aging. (2020) 1:602108. 10.3389/fragi.2020.602108 35822168 PMC9261332

[11] Perch M Hayes D Cherikh WS Zuckermann A Harhay MO Hsich E The international thoracic organ transplant registry of the international society for heart and lung transplantation: thirty-ninth adult lung transplantation report—2022; focus on lung transplant recipients with chronic obstructive pulmonary disease. J Heart Lung Transplant. (2022) 41(10):1335–47. 10.1016/j.healun.2022.08.007 36050206 PMC10257980

[12] Faust HE Golden JA Rajalingam R Wang AS Green G Hays SR Short lung transplant donor telomere length is associated with decreased CLAD-free survival. Thorax. (2017) 72(11):1052–4. 10.1136/thoraxjnl-2016-209897 28446663 PMC6550329

[13] Popescu I Mannem H Winters SA Hoji A Silveira F McNally E Impaired cytomegalovirus immunity in idiopathic pulmonary fibrosis lung transplant recipients with short telomeres. Am J Respir Crit Care Med. (2019) 199(3):362–76. 10.1164/rccm.201805-0825OC 30088779 PMC6363970

[14] Greenland JR Guo R Lee S Tran L Kapse B Kukreja J Short airway telomeres are associated with primary graft dysfunction and chronic lung allograft dysfunction. J Heart Lung Transplant. (2023) 42(12):1700–9. 10.1016/j.healun.2023.08.018 37648073 PMC10858720

[15] Cronkhite JT Xing C Raghu G Chin KM Torres F Rosenblatt RL Telomere shortening in familial and sporadic pulmonary fibrosis. Am J Respir Crit Care Med. (2008) 178(7):729–37. 10.1164/rccm.200804-550OC 18635888 PMC2556455

[16] Bilgili H Białas AJ Górski P Piotrowski WJ. Telomere abnormalities in the pathobiology of idiopathic pulmonary fibrosis. JCM. (2019) 8(8):1232. 10.3390/jcm8081232 31426295 PMC6723768

[17] Rossiello F Jurk D Passos JF d’Adda Di Fagagna F. Telomere dysfunction in ageing and age-related diseases. Nat Cell Biol. (2022) 24(2):135–47. 10.1038/s41556-022-00842-x 35165420 PMC8985209

[18] Bloom S Tuday E Islam MT Gogulamudi V Lesniewski L Donato A. Senolytics reduce endothelial cell DNA damage and telomere dysfunction in old age. Physiology. (2023) 38(S1):5733289. 10.1152/physiol.2023.38.S1.5733289 PMC11922086 40109908

[19] Goronzy JJ Weyand CM. Successful and maladaptive T cell aging. Immunity. (2017) 46(3):364–78. 10.1016/j.immuni.2017.03.010 28329703 PMC5433436

[20] Jacquemont L Tilly G Yap M Doan-Ngoc TM Danger R Guérif P Terminally differentiated effector memory CD8+ T cells identify kidney transplant recipients at high risk of graft failure. J Am Soc Nephrol. (2020) 31(4):876–91. 10.1681/ASN.2019080847 32165419 PMC7191929

[21] Zamora MR Davis RD Leonard C. Management of cytomegalovirus infection in lung transplant recipients: evidence-based recommendations. Transplantation. (2005) 80(2):157–63. 10.1097/01.TP.0000165430.65645.4F 16041258

[22] Ravkov E Slev P Heikal N. Thymic output: assessment of CD4+ recent thymic emigrants and T-cell receptor excision circles in infants: THYMIC OUTPUT. Cytometry. (2017) 92(4):249–57. 10.1002/cyto.b.21341 26566232

[23] Mitchell WA Lang PO Aspinall R. Tracing thymic output in older individuals. Clin Exp Immunol. (2010) 161(3):497–503. 10.1111/j.1365-2249.2010.04209.x 20646007 PMC2962967

[24] Courivaud C Bamoulid J Crepin T Gaiffe E Laheurte C Saas P Pre-transplant thymic function predicts is associated with patient death after kidney transplantation. Front Immunol. (2020) 11:1653. 10.3389/fimmu.2020.01653 32903778 PMC7438875

[25] Söderström A Vonlanthen S Jönsson-Videsäter K Mielke S Lindahl H Törlén J T cell receptor excision circles are potential predictors of survival in adult allogeneic hematopoietic stem cell transplantation recipients with acute myeloid leukemia. Front Immunol. (2022) 13:954716. 10.3389/fimmu.2022.954716 36211398 PMC9540498

[26] Gracia-Ahufinger I Ferrando-Martínez S Montejo M Muñoz-Villanueva MC Cantisán S Rivero A Pre-transplant thymic function is associated with the risk of cytomegalovirus disease after solid organ transplantation. Clin Microbiol Infect. (2015) 21(5):511.e1–e7. 10.1016/j.cmi.2014.12.020 25682299

[27] Buckley CE Dorsey FC. The effect of aging on human serum immunoglobulin concentrations. J Immunol. (1970) 105(4):964–72. 10.4049/jimmunol.105.4.964 4097147

[28] Florescu DF Kalil AC Qiu F Schmidt CM Sandkovsky U. What is the impact of hypogammaglobulinemia on the rate of infections and survival in solid organ transplantation? A meta-analysis. Am J Transplant. (2013) 13(10):2601–10. 10.1111/ajt.12401 23919557

[29] Otani IM Lehman HK Jongco AM Tsao LR Azar AE Tarrant TK Practical guidance for the diagnosis and management of secondary hypogammaglobulinemia: a work group report of the AAAAI primary immunodeficiency and altered immune response committees. J Allergy Clin Immunol. (2022) 149(5):1525–60. 10.1016/j.jaci.2022.01.025 35176351

[30] Goldfarb NS Avery RK Goormastic M Mehta AC Schilz R Smedira N Hypogammaglobulinemia in lung transplant recipients. Transplantation. (2001) 71(2):242–6. 10.1097/00007890-200101270-00013 11213067

[31] Passos JF Saretzki G Ahmed S Nelson G Richter T Peters H Mitochondrial dysfunction accounts for the stochastic heterogeneity in telomere-dependent senescence. De lange T, editor. PLoS Biol. (2007) 5(5):e110. 10.1371/journal.pbio.0050110 17472436 PMC1858712

[32] Ferri E Marzetti E Calvani R Picca A Cesari M Arosio B. Role of age-related mitochondrial dysfunction in sarcopenia. Int J Mol Sci. (2020) 21(15):5236. 10.3390/ijms21155236 32718064 PMC7432902

[33] BeckwéeDDelaereAAelbrechtSBaertVBeaudartCBruyereO Exercise interventions for the prevention and treatment of sarcopenia. A systematic Umbrella review. J Nutr Health Aging. (2019) 23(6):494–502. 10.1007/s12603-019-1196-831233069

[34] Scozzi D Ibrahim M Liao F Lin X Hsiao HM Hachem R Mitochondrial damage–associated molecular patterns released by lung transplants are associated with primary graft dysfunction. Am J Transplant. (2019) 19(5):1464–77. 10.1111/ajt.15232 30582269 PMC6482093

[35] Iske J Seyda M Heinbokel T Maenosono R Minami K Nian Y Senolytics prevent mt-DNA-induced inflammation and promote the survival of aged organs following transplantation. Nat Commun. (2020) 11(1):4289. 10.1038/s41467-020-18039-x 32855397 PMC7453018

[36] Jang YJ. The effects of protein and supplements on sarcopenia in human clinical studies: how older adults should consume protein and supplements. J Microbiol Biotechnol. (2023) 33(2):143–50. 10.4014/jmb.2210.10014 36474318 PMC9998208

[37] Torii M Itaya T Minamino H Katsushima M Fujita Y Tanaka H Management of sarcopenia in patients with rheumatoid arthritis. Modern Rheumatology. (2023) 33(3):435–40. 10.1093/mr/roac095 35986513

[38] Gruber HJ Semeraro MD Renner W Herrmann M. Telomeres and age-related diseases. Biomedicines. (2021) 9(10):1335. 10.3390/biomedicines9101335 34680452 PMC8533433

[39] Lu W Zhang Y Liu D Songyang Z Wan M. Telomeres—structure, function, and regulation. Exp Cell Res. (2013) 319(2):133–41. 10.1016/j.yexcr.2012.09.005 23006819 PMC4051234

[40] Liu Y Snow BE Hande MP Yeung D Erdmann NJ Wakeham A The telomerase reverse transcriptase is limiting and necessary for telomerase function in vivo. Curr Biol. (2000) 10(22):1459–62. 10.1016/S0960-9822(00)00805-8 11102810

[41] Blasco MA Lee HW Hande MP Samper E Lansdorp PM DePinho RA Telomere shortening and tumor formation by mouse cells lacking telomerase RNA. Cell. (1997) 91(1):25–34. 10.1016/S0092-8674(01)80006-4 9335332

[42] Boccardi M Boccardi V. Psychological wellbeing and healthy aging: focus on telomeres. Geriatrics. (2019) 4(1):25. 10.3390/geriatrics4010025 31023993 PMC6473912

[43] Gao Z Daquinag AC Fussell C Zhao Z Dai Y Rivera A Age-associated telomere attrition in adipocyte progenitors predisposes to metabolic disease. Nat Metab. (2020) 2(12):1482–97. 10.1038/s42255-020-00320-4 33324010

[44] Bhattacharyya J Mihara K Bhattacharjee D Mukherjee M. Telomere length as a potential biomarker of coronary artery disease. Indian J Med Res. (2017) 145(6):730. 10.4103/0971-5916.216974 29067974 PMC5674542

[45] Thomas P O’ Callaghan NJ Fenech M. Telomere length in white blood cells, buccal cells and brain tissue and its variation with ageing and Alzheimer’s disease. Mech Ageing Dev. (2008) 129(4):183–90. 10.1016/j.mad.2007.12.004 18242664

[46] Valdes AM Richards JB Gardner JP Swaminathan R Kimura M Xiaobin L Telomere length in leukocytes correlates with bone mineral density and is shorter in women with osteoporosis. Osteoporos Int. (2007) 18(9):1203–10. 10.1007/s00198-007-0357-5 17347788

[47] Fordyce CA Heaphy CM Bisoffi M Wyaco JL Joste NE Mangalik A Telomere content correlates with stage and prognosis in breast cancer. Breast Cancer Res Treat. (2006) 99(2):193–202. 10.1007/s10549-006-9204-1 16752076

[48] McNally EJ Luncsford PJ Armanios M. Long telomeres and cancer risk: the price of cellular immortality. J Clin Invest. (2019) 129(9):3474–81. 10.1172/JCI120851 31380804 PMC6715353

[49] Herbig U Jobling WA Chen BPC Chen DJ Sedivy JM. Telomere shortening triggers senescence of human cells through a pathway involving ATM, p53, and p21CIP1, but not p16INK4a. Mol Cell. (2004) 14(4):501–13. 10.1016/S1097-2765(04)00256-4 15149599

[50] Sławińska N Krupa R. Molecular aspects of senescence and organismal ageing-DNA damage response, telomeres, inflammation and chromatin. Int J Mol Sci. (2021) 22(2):590. 10.3390/ijms22020590 33435578 PMC7827783

[51] Opresko PL Shay JW. Telomere-associated aging disorders. Ageing Res Rev. (2017) 33:52–66. 10.1016/j.arr.2016.05.009 27215853 PMC9926533

[52] Holohan B Wright WE Shay JW. Telomeropathies: an emerging spectrum disorder. J Cell Biol. (2014) 205(3):289–99. 10.1083/jcb.201401012 24821837 PMC4018777

[53] Kesäniemi J Lavrinienko A Tukalenko E Boratyński Z Kivisaari K Mappes T Exposure to environmental radionuclides associates with tissue-specific impacts on telomerase expression and telomere length. Sci Rep. (2019) 9(1):850. 10.1038/s41598-018-37164-8 30696885 PMC6351625

[54] Duan R Fu Q Sun Y Li Q. Epigenetic clock: a promising biomarker and practical tool in aging. Ageing Res Rev. (2022) 81:101743. 10.1016/j.arr.2022.101743 36206857

[55] Kressler C Gasparoni G Nordström K Hamo D Salhab A Dimitropoulos C Targeted De-methylation of the FOXP3-TSDR is sufficient to induce physiological FOXP3 expression but not a functional treg phenotype. Front Immunol. (2021) 11:609891. 10.3389/fimmu.2020.609891 33488615 PMC7817622

[56] Lu AT Fei Z Haghani A Robeck TR Zoller JA Li CZ Author correction: universal DNA methylation age across mammalian tissues. Nat Aging. (2023) 3(11):1462. 10.1038/s43587-023-00499-7 37674040 PMC10645586

[57] Horvath S. DNA methylation age of human tissues and cell types. Genome Biol. (2013) 14(10):R115. 10.1186/gb-2013-14-10-r115 24138928 PMC4015143

[58] Higgins-Chen AT Boks MP Vinkers CH Kahn RS Levine ME. Schizophrenia and epigenetic aging biomarkers: increased mortality, reduced cancer risk, and unique clozapine effects. Biol Psychiatry. (2020) 88(3):224–35. 10.1016/j.biopsych.2020.01.025 32199607 PMC7368835

[59] Fiorito G Caini S Palli D Bendinelli B Saieva C Ermini I DNA methylation-based biomarkers of aging were slowed down in a two-year diet and physical activity intervention trial: the DAMA study. Aging Cell. (2021) 20(10):e13439. 10.1111/acel.13439 34535961 PMC8520727

[60] Harvanek ZM Boks MP Vinkers CH Higgins-Chen AT. The cutting edge of epigenetic clocks: in search of mechanisms linking aging and mental health. Biol Psychiatry. (2023) 94(9):694–705. 10.1016/j.biopsych.2023.02.001 36764569 PMC10409884

[61] Schaenman J Zhou X Guo R Rossetti M Liang EC Lum E DNA methylation age is more closely associated with infection risk than chronological age in kidney transplant recipients. Transplant Direct. (2020) 6(8):e576. 10.1097/TXD.0000000000001020 33134500 PMC7581059

[62] Soegiarto G Purnomosari D. Challenges in the vaccination of the elderly and strategies for improvement. Pathophysiology. (2023) 30(2):155–73. 10.3390/pathophysiology30020014 37218912 PMC10204411

[63] Kwok JSY Cheung SKF Ho JCY Tang IWH Chu PWK Leung EYS Establishing simultaneous T cell receptor excision circles (TREC) and K-deleting recombination excision circles (KREC) quantification assays and laboratory reference intervals in healthy individuals of different age groups in Hong Kong. Front Immunol. (2020) 11:1411. 10.3389/fimmu.2020.01411 32765500 PMC7378446

[64] Takeshita S Toda M Yamagishi H. Excision products of the T cell receptor gene support a progressive rearrangement model of the alpha/delta locus. EMBO J. (1989) 8(11):3261–70. 10.1002/j.1460-2075.1989.tb08486.x 2583098 PMC401453

[65] Miller JFAP. Effect of neonatal thymectomy on the immunological responsiveness of the mouse. Proc R Soc Lond B. (1962) 156(964):415–28. 10.1098/rspb.1962.0048

[66] Miller JFAP. Role of the thymus in transplantation immunity*. Ann N Y Acad Sci. (2006) 99(3):340–54. 10.1111/j.1749-6632.1962.tb45319.x

[67] Miller JFAP. The golden anniversary of the thymus. Nat Rev Immunol. (2011) 11(7):489–95. 10.1038/nri2993 21617694

[68] Lynch HE Goldberg GL Chidgey A Van Den Brink MRM Boyd R Sempowski GD. Thymic involution and immune reconstitution. Trends Immunol. (2009) 30(7):366–73. 10.1016/j.it.2009.04.003 19540807 PMC2750859

[69] Wertheimer AM Bennett MS Park B Uhrlaub JL Martinez C Pulko V Aging and cytomegalovirus infection differentially and jointly affect distinct circulating T cell subsets in humans. J Immunol. (2014) 192(5):2143–55. 10.4049/jimmunol.1301721 24501199 PMC3989163

[70] Schwarz C Mahr B Muckenhuber M Weijler AM Unger LW Pilat N In vivo treg expansion under costimulation blockade targets early rejection and improves long-term outcome. Am J Transplant. (2021) 21(11):3765–74. 10.1111/ajt.16724 34152692 PMC9292010

[71] Khan MA Lau CL Krupnick AS. Monitoring regulatory T cells as a prognostic marker in lung transplantation. Front Immunol. (2023) 14:1235889. 10.3389/fimmu.2023.1235889 37818354 PMC10561299

[72] Natalini JG Singh S Segal LN. The dynamic lung microbiome in health and disease. Nat Rev Microbiol. (2023) 21(4):222–35. 10.1038/s41579-022-00821-x 36385637 PMC9668228

[73] Tang Q Leung J Peng Y Sanchez-Fueyo A Lozano JJ Lam A Selective decrease of donor-reactive tregs after liver transplantation limits treg therapy for promoting allograft tolerance in humans. Sci Transl Med. (2022) 14(669):eabo2628. 10.1126/scitranslmed.abo2628 36322627 PMC11016119

[74] Muller-Sieburg CE Sieburg HB Bernitz JM Cattarossi G. Stem cell heterogeneity: implications for aging and regenerative medicine. Blood. (2012) 119(17):3900–7. 10.1182/blood-2011-12-376749 22408258 PMC3355711

[75] Anspach J Poulsen G Kaattari I Pollock R Zwollo P. Reduction in DNA binding activity of the transcription factor pax-5a in B lymphocytes of aged mice. J Immunol. (2001) 166(4):2617–26. 10.4049/jimmunol.166.4.2617 11160324

[76] Stephan RP Reilly CR Witte PL. Impaired ability of bone marrow stromal cells to support B-lymphopoiesis with age. Blood. (1998) 91(1):75–88. 10.1182/blood.V91.1.75 9414271

[77] Frasca D Van Der Put E Riley RL Blomberg BB. Reduced ig class switch in aged mice correlates with decreased E47 and activation-induced cytidine deaminase. J Immunol. (2004) 172(4):2155–62. 10.4049/jimmunol.172.4.2155 14764681

[78] Cancro MP. Age-associated B cells. Annu Rev Immunol. (2020) 38(1):315–40. 10.1146/annurev-immunol-092419-031130 31986068

[79] Mori DN Shen H Galan A Goldstein DR. Aged B cells alter immune regulation of allografts in mice. Eur J Immunol. (2016) 46(11):2650–8. 10.1002/eji.201646353 27546296 PMC5366259

[80] Roh JS Sohn DH. Damage-associated molecular patterns in inflammatory diseases. Immune Netw. (2018) 18(4):e27. 10.4110/in.2018.18.e27 30181915 PMC6117512

[81] McGovern KE Sonar SA Watanabe M Coplen CP Bradshaw CM Nikolich JŽ. The aging of the immune system and its implications for transplantation. Geroscience. (2023) 45:1383–400. 10.1007/s11357-022-00720-2 36626019 PMC9838392

[82] Woo YD Jeong D Chung DH. Development and functions of alveolar macrophages. MolCells. (2021) 44(5):292–300. 10.14348/molcells.2021.0058 PMC8175155 33972474

[83] De Maeyer RPH Chambers ES. The impact of ageing on monocytes and macrophages. Immunol Lett. (2021) 230:1–10. 10.1016/j.imlet.2020.12.003 33309673

[84] Scalea JR Lee YS Davila E Bromberg JS. Myeloid-derived suppressor cells and their potential application in transplantation. Transplantation. (2018) 102(3):359–67. 10.1097/TP.0000000000002022 29189485 PMC6890230

[85] Schroeter A Roesel MJ Matsunaga T Xiao Y Zhou H Tullius SG. Aging affects the role of myeloid-derived suppressor cells in alloimmunity. Front Immunol. (2022) 13:917972. 10.3389/fimmu.2022.917972 35874716 PMC9296838

[86] Baixauli F Acín-Pérez R Villarroya-Beltrí C Mazzeo C Nuñez-Andrade N Gabandé-Rodriguez E Mitochondrial respiration controls lysosomal function during inflammatory T cell responses. Cell Metab. (2015) 22(3):485–98. 10.1016/j.cmet.2015.07.020 26299452 PMC5026297

[87] Ramstead AG Wallace JA Lee SH Bauer KM Tang WW Ekiz HA Mitochondrial pyruvate carrier 1 promotes peripheral T cell homeostasis through metabolic regulation of thymic development. Cell Rep. (2020) 30(9):2889–2899.e6. 10.1016/j.celrep.2020.02.042 32130894 PMC7170217

[88] Martínez-Reyes I Chandel NS. Mitochondrial TCA cycle metabolites control physiology and disease. Nat Commun. (2020) 11(1):102. 10.1038/s41467-019-13668-3 31900386 PMC6941980

[89] Nolfi-Donegan D Braganza A Shiva S. Mitochondrial electron transport chain: oxidative phosphorylation, oxidant production, and methods of measurement. Redox Biol. (2020) 37:101674. 10.1016/j.redox.2020.101674 32811789 PMC7767752

[90] Hutter E Renner K Pfister G Stöckl P Jansen-Dürr P Gnaiger E. Senescence-associated changes in respiration and oxidative phosphorylation in primary human fibroblasts. Biochem J. (2004) 380(3):919–28. 10.1042/bj20040095 15018610 PMC1224220

[91] Heo S Kim S Kang D. The role of hydrogen peroxide and peroxiredoxins throughout the cell cycle. Antioxidants (Basel). (2020) 9(4):280. 10.3390/antiox9040280 32224940 PMC7222192

[92] Chen P Hu YF Wang L Xiao WF Bao XY Pan C Mitochondrial apoptotic pathway is activated by H_2_O_2_-mediated oxidative stress in BmN-SWU1 cells from bombyx mori ovary. PLoS One. (2015) 10(7):e0134694. 10.1371/journal.pone.0134694 26225758 PMC4520666

[93] Lee S Tak E Lee J Rashid M Murphy MP Ha J Mitochondrial H_2_O_2_ generated from electron transport chain complex I stimulates muscle differentiation. Cell Res. (2011) 21(5):817–34. 10.1038/cr.2011.55 21445095 PMC3203677

[94] Chen Q Fischer A Reagan JD Yan LJ Ames BN. Oxidative DNA damage and senescence of human diploid fibroblast cells. Proc Natl Acad Sci USA. (1995) 92(10):4337–41. 10.1073/pnas.92.10.4337 7753808 PMC41939

[95] Passos JF Nelson G Wang C Richter T Simillion C Proctor CJ Feedback between p21 and reactive oxygen production is necessary for cell senescence. Mol Syst Biol. (2010) 6(1):347. 10.1038/msb.2010.5 20160708 PMC2835567

[96] Zhang W Wang M Xie HY Zhou L Meng XQ Shi J Role of reactive oxygen species in mediating hepatic ischemia-reperfusion injury and its therapeutic applications in liver transplantation. Transplant Proc. (2007) 39(5):1332–7. 10.1016/j.transproceed.2006.11.021 17580134

[97] Rosenberg IH. Sarcopenia: origins and clinical relevance. J Nutr. (1997) 127(5):990S–1S. 10.1093/jn/127.5.990S 9164280

[98] Daussin FN Boulanger E Lancel S. From mitochondria to sarcopenia: role of inflammaging and RAGE-ligand axis implication. Exp Gerontol. (2021) 146:111247. 10.1016/j.exger.2021.111247 33484891

[99] Shen Y Shi Q Nong K Li S Yue J Huang J Exercise for sarcopenia in older people: a systematic review and network meta-analysis. J Cachexia Sarcopenia Muscle. (2023) 14(3):1199–211. 10.1002/jcsm.13225 37057640 PMC10235889

[100] Minhas PS Latif-Hernandez A McReynolds MR Durairaj AS Wang Q Rubin A Restoring metabolism of myeloid cells reverses cognitive decline in ageing. Nature. (2021) 590(7844):122–8. 10.1038/s41586-020-03160-0 33473210 PMC8274816

[101] Bouviere J Fortunato RS Dupuy C Werneck-de-Castro JP Carvalho DP Louzada RA. Exercise-stimulated ROS sensitive signaling pathways in skeletal muscle. Antioxidants. (2021) 10(4):537. 10.3390/antiox10040537 33808211 PMC8066165

[102] Vasilaki A Mansouri A Van Remmen H Van Der Meulen JH Larkin L Richardson AG Free radical generation by skeletal muscle of adult and old mice: effect of contractile activity. Aging Cell. (2006) 5(2):109–17. 10.1111/j.1474-9726.2006.00198.x 16626390

[103] Powers SK Goldstein E Schrager M Ji LL. Exercise training and skeletal muscle antioxidant enzymes: an update. Antioxidants. (2022) 12(1):39. 10.3390/antiox12010039 36670901 PMC9854578

[104] Fan Z Yang JY Guo Y Liu YX Zhong XY. Altered levels of circulating mitochondrial DNA in elderly people with sarcopenia: association with mitochondrial impairment. Exp Gerontol. (2022) 163:111802. 10.1016/j.exger.2022.111802 35398474

[105] Nakahira K Haspel JA Rathinam VAK Lee SJ Dolinay T Lam HC Autophagy proteins regulate innate immune responses by inhibiting the release of mitochondrial DNA mediated by the NALP3 inflammasome. Nat Immunol. (2011) 12(3):222–30. 10.1038/ni.1980 21151103 PMC3079381

[106] White MJ McArthur K Metcalf D Lane RM Cambier JC Herold MJ Apoptotic caspases suppress mtDNA-induced STING-mediated type I IFN production. Cell. (2014) 159(7):1549–62. 10.1016/j.cell.2014.11.036 25525874 PMC4520319

[107] Lee JC Christie JD. Primary graft dysfunction. Clin Chest Med. (2011) 32(2):279–93. 10.1016/j.ccm.2011.02.007 21511090

[108] Fulle S Protasi F Di Tano G Pietrangelo T Beltramin A Boncompagni S The contribution of reactive oxygen species to sarcopenia and muscle ageing. Exp Gerontol. (2004) 39(1):17–24. 10.1016/j.exger.2003.09.012 14724060

[109] Rael VE Chen L McIntosh CM Alegre ML. Exercise increases skin graft resistance to rejection. Am J Transplant. (2019) 19(5):1560–7. 10.1111/ajt.15266 30659772 PMC7137356

[110] Suzuki K Tominaga T Ruhee RT Ma S. Characterization and modulation of systemic inflammatory response to exhaustive exercise in relation to oxidative stress. Antioxidants. (2020) 9(5):401. 10.3390/antiox9050401 32397304 PMC7278761

[111] Liao P He Q Zhou X Ma K Wen J Chen H Repetitive bouts of exhaustive exercise induces a systemic inflammatory response and multi-organ damage in rats. Front Physiol. (2020) 11:685. 10.3389/fphys.2020.00685 32655413 PMC7324715

[112] Conte M Martucci M Chiariello A Franceschi C Salvioli S. Mitochondria, immunosenescence and inflammaging: a role for mitokines? Semin Immunopathol. (2020) 42(5):607–17. 10.1007/s00281-020-00813-0 32757036 PMC7666292

[113] Schratz KE. Extrahematopoietic manifestations of the short telomere syndromes. Hematology. (2020) 1:115–22. 10.1182/hematology.2020000170 PMC7727508 33275732

[114] Silhan LL Shah PD Chambers DC Snyder LD Riise GC Wagner CL Lung transplantation in telomerase mutation carriers with pulmonary fibrosis. Eur Respir J. (2014) 44(1):178–87. 10.1183/09031936.00060014 24833766 PMC4076528

[115] Alder JK Sutton RM Iasella CJ Nouraie M Koshy R Hannan SJ Lung transplantation for idiopathic pulmonary fibrosis enriches for individuals with telomere-mediated disease. J Heart Lung Transplant. (2022) 41(5):654–63. 10.1016/j.healun.2021.11.008 34933798 PMC9038609

[116] Newton CA Kozlitina J Lines JR Kaza V Torres F Garcia CK. Telomere length in patients with pulmonary fibrosis associated with chronic lung allograft dysfunction and post–lung transplantation survival. J Heart Lung Transplant. (2017) 36(8):845–53. 10.1016/j.healun.2017.02.005 28262440 PMC5515686

[117] Banaszak LG Smith-Simmer K Shoger K Lovrien L Malik A Sandbo N Implementation of a prospective screening strategy to identify adults with a telomere biology disorder among those undergoing lung transplant evaluation for interstitial lung disease. Respir Med. (2023) 220:107464. 10.1016/j.rmed.2023.107464 37951311

[118] Courtwright AM Lamattina AM Takahashi M Trindade AJ Hunninghake GM Rosas IO Shorter telomere length following lung transplantation is associated with clinically significant leukopenia and decreased chronic lung allograft dysfunction-free survival. ERJ Open Res. (2020) 6(2):00003–2020. 10.1183/23120541.00003-2020 32577419 PMC7293991

[119] Snyder ME Anderson MR Benvenuto LJ Sutton RM Bondonese A Koshy R Impact of age and telomere length on circulating T cells and rejection risk after lung transplantation for idiopathic pulmonary fibrosis. J Heart Lung Transplant. (2023) 42(12):1666–77. 10.1016/j.healun.2023.08.001 37544465 PMC10839116

[120] Trzonkowski P Dębska-Ślizień A Jankowska M Wardowska A Carvalho-Gaspar M Hak Ł Immunosenescence increases the rate of acceptance of kidney allotransplants in elderly recipients through exhaustion of CD4+ T-cells. Mech Ageing Dev. (2010) 131(2):96–104. 10.1016/j.mad.2009.12.006 20060852

[121] Wang P Leung J Lam A Lee S Calabrese DR Hays SR Lung transplant recipients with idiopathic pulmonary fibrosis have impaired alloreactive immune responses. J Heart Lung Transplant. (2022) 41(5):641–53. 10.1016/j.healun.2021.11.012 34924263 PMC9038662

[122] Iske J Dedeilia A Xiao Y Martin F Emmert MY Sage PT The impact of T-cell aging on alloimmunity and inflammaging. Transplantation. (2023). 10.1097/TP.0000000000004715. [Epub ahead of print] 37389638 PMC10756935

[123] Meier-Kriesche HU Ojo AO Cibrik DM Hanson JA Leichtman AB Magee JC Relationship of recipient age and development of chronic allograft failure. Transplantation. (2000) 70(2):306–10. 10.1097/00007890-200007270-00012 10933154

[124] Nandavaram S Chandrashekaran S Gelman AE. Short telomeres in lung transplantation: known unknowns. J Heart Lung Transplant. (2022) 41(5):664–6. 10.1016/j.healun.2022.02.001 35351386

[125] Steel JC Di Pasquale G Ramlogan CA Patel V Chiorini JA Morris JC. Oral vaccination with adeno-associated virus vectors expressing the neu oncogene inhibits the growth of murine breast cancer. Mol Ther. (2013) 21(3):680–7. 10.1038/mt.2012.260 23295951 PMC3589150

[126] Hannan SJ Iasella CJ Sutton RM Popescu ID Koshy R Burke R Lung transplant recipients with telomere-mediated pulmonary fibrosis have increased risk for hematologic complications. Am J Transplant. (2023) 23(10):1590–602. 10.1016/j.ajt.2023.06.014 37392813 PMC11062487

[127] Tague LK Scozzi D Wallendorf M Gage BF Krupnick AS Kreisel D Lung transplant outcomes are influenced by severity of neutropenia and granulocyte colony-stimulating factor treatment. Am J Transplant. (2020) 20(1):250–61. 10.1111/ajt.15581 31452317 PMC6940547

[128] Wagner CL Hanumanthu VS Talbot CC Abraham RS Hamm D Gable DL Short telomere syndromes cause a primary T cell immunodeficiency. J Clin Invest. (2018) 128(12):5222–34. 10.1172/JCI120216 30179220 PMC6264634

[129] Snyder LD Finlen-Copeland CA Turbyfill WJ Howell D Willner DA Palmer SM. Cytomegalovirus pneumonitis is a risk for bronchiolitis obliterans syndrome in lung transplantation. Am J Respir Crit Care Med. (2010) 181(12):1391–6. 10.1164/rccm.200911-1786OC 20167845 PMC2894412

[130] Saullo JL Baker AW Snyder LD Reynolds JM Zaffiri L Eichenberger EM Cytomegalovirus prevention in thoracic organ transplantation: a single-center evaluation of letermovir prophylaxis. J Heart Lung Transplant. (2022) 41(4):508–15. 10.1016/j.healun.2021.12.005 35031206 PMC9121640

[131] Iasella CJ Winters SA Kois A Cho J Hannan SJ Koshy R Idiopathic pulmonary fibrosis lung transplant recipients are at increased risk for EBV-associated posttransplant lymphoproliferative disorder and worse survival. Am J Transplant. (2020) 20(5):1439–46. 10.1111/ajt.15756 31874120 PMC8130541

[132] Calado RT Regal JA Kleiner DE Schrump DS Peterson NR Pons V A Spectrum of severe familial liver disorders associate with telomerase mutations. Klein R, editor. PLoS ONE. (2009) 4(11):e7926. 10.1371/journal.pone.0007926 19936245 PMC2775683

[133] Gorgy AI Jonassaint NL Stanley SE Koteish A DeZern AE Walter JE Hepatopulmonary syndrome is a frequent cause of dyspnea in the short telomere disorders. Chest. (2015) 148(4):1019–26. 10.1378/chest.15-0825 26158642 PMC4594621

[134] Moschouri E Vionnet J Giostra E Daccord C Lazor R Sciarra A Combined lung and liver transplantation for short telomere syndrome. Liver Transpl. (2020) 26(6):840–4. 10.1002/lt.25734 32080954

[135] Lebeer M Wuyts WA Cassiman D Laleman W Nevens F Pirenne J Multiple solid organ transplantation in telomeropathy: case series and literature review. Transplantation. (2018) 102(10):1747–55. 10.1097/TP.0000000000002198 29596117

[136] Bleve A Motta F Durante B Pandolfo C Selmi C Sica A. Immunosenescence, inflammaging, and frailty: role of myeloid cells in age-related diseases. Clinic Rev Allerg Immunol. (2022) 64(2):123–44. 10.1007/s12016-021-08909-7 PMC8760106 35031957

[137] Schratz KE Armanios M. Cancer and myeloid clonal evolution in the short telomere syndromes. Curr Opin Genet Dev. (2020) 60:112–8. 10.1016/j.gde.2020.02.019 32276199 PMC8122241

[138] McDyer JF McIntyre S Chen X Koshy R Popescu I Stanczak H Tandem bilateral lung transplantation and bone marrow transplantation in select patients with End-stage lung disease: the potential for allograft acceptance and immunosuppression withdrawal. A15 LUNG TRANSPLANTATION I (2020), American Thoracic Society. p. A1021–A1021. Available online at: https://www.atsjournals.org/doi/10.1164/ajrccm-conference.2020.201.1_MeetingAbstracts.A1021 (cited November 16, 2023).

[139] Dugger DT Calabrese DR Gao Y Deiter F Tsao T Maheshwari J Lung allograft epithelium DNA methylation age is associated with graft chronologic age and primary graft dysfunction. Front Immunol. (2021) 12:704172. 10.3389/fimmu.2021.704172 34691018 PMC8528961

[140] Naikawadi RP Green G Jones KD Achtar-Zadeh N Mieleszko JE Arnould I Airway epithelial telomere dysfunction drives remodeling similar to chronic lung allograft dysfunction. Am J Respir Cell Mol Biol. (2020) 63(4):490–501. 10.1165/rcmb.2019-0374OC 32551854 PMC7528921

[141] Courtwright AM Fried S Villalba JA Moniodis A Guleria I Wood I Association of donor and recipient telomere length with clinical outcomes following lung transplantation. PLoS One. (2016) 11(9):e0162409. 10.1371/journal.pone.0162409 27589328 PMC5010211

[142] De Vlaminck I Martin L Kertesz M Patel K Kowarsky M Strehl C Noninvasive monitoring of infection and rejection after lung transplantation. Proc Natl Acad Sci USA. (2015) 112(43):13336–41. 10.1073/pnas.1517494112 26460048 PMC4629384

[143] Abbasi J. Researchers tie severe immunosuppression to chronic COVID-19 and virus variants. JAMA. (2021) 325(20):2033. 10.1001/jama.2021.7212 33950236

[144] Hallett AM Greenberg RS Boyarsky BJ Shah PD Ou MT Teles AT SARS-CoV-2 messenger RNA vaccine antibody response and reactogenicity in heart and lung transplant recipients. J Heart Lung Transplant. (2021) 40(12):1579–88. 10.1016/j.healun.2021.07.026 34456108 PMC8349311

[145] Sindu D Razia D Bay C Padiyar J Grief K Buddhdev B Evolving impact of the COVID-19 pandemic on lung transplant recipients: a single-center experience. J Heart Lung Transplant. (2023):S1053-2498(23)02073-9. [Epub ahead of print] 37852512 10.1016/j.healun.2023.10.010

[146] Frey S Ruck JM Alejo JL Barker L Matthew J Werbel WA Perivaccination antimetabolite hold and third dose of SARS-CoV-2 vaccine in lung transplant recipients: preliminary report. Transplantation. (2022) 106(9):e426–8. 10.1097/TP.0000000000004240 35698264 PMC10123514

[147] Greenland JR Letvin NL. Chemical adjuvants for plasmid DNA vaccines. Vaccine. (2007) 25(19):3731–41. 10.1016/j.vaccine.2007.01.120 17350735

[148] Geiben-Lynn R Greenland JR Frimpong-Boateng K Letvin NL. Kinetics of recombinant adenovirus type 5, vaccinia virus, modified vaccinia Ankara virus, and DNA antigen expression in vivo and the induction of memory T-lymphocyte responses. Clin Vaccine Immunol. (2008) 15(4):691–6. 10.1128/CVI.00418-07 18272665 PMC2292663

[149] Singer JP Lederer DJ Baldwin MR. Frailty in pulmonary and critical care medicine. Annals ATS. (2016) 13(8):1394–404. 10.1513/AnnalsATS.201512-833FR PMC5021078 27104873

[150] Venado A Kolaitis NA Huang CY Gao Y Glidden DV Soong A Frailty after lung transplantation is associated with impaired health-related quality of life and mortality. Thorax. (2020) 75(8):669–78. 10.1136/thoraxjnl-2019-213988 32376733 PMC8023537

[151] Singer JP Gao Y Huang CY Kordahl RC Sriram A Hays SR The association between frailty and chronic lung allograft dysfunction after lung transplantation. Transplantation. (2023) 107(10):2255–61. 10.1097/TP.0000000000004672 37287095 PMC10524113

[152] Singer JP Calfee CS Delucchi K Diamond JM Anderson MA Benvenuto LA Subphenotypes of frailty in lung transplant candidates. Am J Transplant. (2023) 23(4):531–9. 10.1016/j.ajt.2023.01.020 36740192 PMC11005295

[153] Bountziouka V Nelson CP Codd V Wang Q Musicha C Allara E Association of shorter leucocyte telomere length with risk of frailty. J Cachexia Sarcopenia Muscle. (2022) 13(3):1741–51. 10.1002/jcsm.12971 35297226 PMC9178164

[154] Singer JP Soong A Bruun A Bracha A Chin G Hays SR A mobile health technology enabled home-based intervention to treat frailty in adult lung transplant candidates: a pilot study. Clin Transplant. (2018) 32(6):e13274. 10.1111/ctr.13274 29742287 PMC6066279

[155] Sellami M Gasmi M Denham J Hayes LD Stratton D Padulo J Effects of acute and chronic exercise on immunological parameters in the elderly aged: can physical activity counteract the effects of aging? Front Immunol. (2018) 9:2187. 10.3389/fimmu.2018.02187 30364079 PMC6191490

[156] Jacobson PA Schladt D Oetting WS Leduc R Guan W Matas AJ Lower calcineurin inhibitor doses in older compared to younger kidney transplant recipients yield similar troughs. Am J Transplant. (2012) 12(12):3326–36. 10.1111/j.1600-6143.2012.04232.x 22947444 PMC3513646

[157] Falck P Åsberg A Byberg KT Bremer S Bergan S Reubsaet JLE Reduced elimination of cyclosporine A in elderly (>65 years) kidney transplant recipients. Transplantation. (2008) 86(10):1379–83. 10.1097/TP.0b013e31818aa4b6 19034006

[158] Stuck AE Frey BM Frey FJ. Kinetics of prednisolone and endogenous cortisol suppression in the elderly. Clin Pharmacol Ther. (1988) 43(4):354–62. 10.1038/clpt.1988.43 3356080

[159] Krenzien F Quante M Heinbokel T Seyda M Minami K Uehara H Age-Dependent metabolic and immunosuppressive effects of tacrolimus. Am J Transplant. (2017) 17(5):1242–54. 10.1111/ajt.14087 27754593 PMC5395364

[160] Mannick JB Del Giudice G Lattanzi M Valiante NM Praestgaard J Huang B mTOR inhibition improves immune function in the elderly. Sci Transl Med. (2014) 6(268):268ra179. 10.1126/scitranslmed.3009892 25540326

[161] Leard LE Holm AM Valapour M Glanville AR Attawar S Aversa M Consensus document for the selection of lung transplant candidates: an update from the international society for heart and lung transplantation. J Heart Lung Transplant. (2021) 40(11):1349–79. 10.1016/j.healun.2021.07.005 34419372 PMC8979471

